# Supplementary material for: A Humanized Yeast Phenomic Model of Deoxycytidine Kinase to Predict Genetic Buffering of Nucleoside Analog Cytotoxicity
Source: Genes (Basel). 2019 Sep 30;10(10):770. doi: 10.3390/genes10100770 (PMC6826991; doi:10.3390/genes10100770)
Supplement: Supplementary file 1 [file genes-10-00770-s001.zip › Additional_Files/Additional_File7_GOTermHeatmaps.zip!/Additional_File7_GOTermHeatmaps/nucleocytoplasmic_transport.pdf]

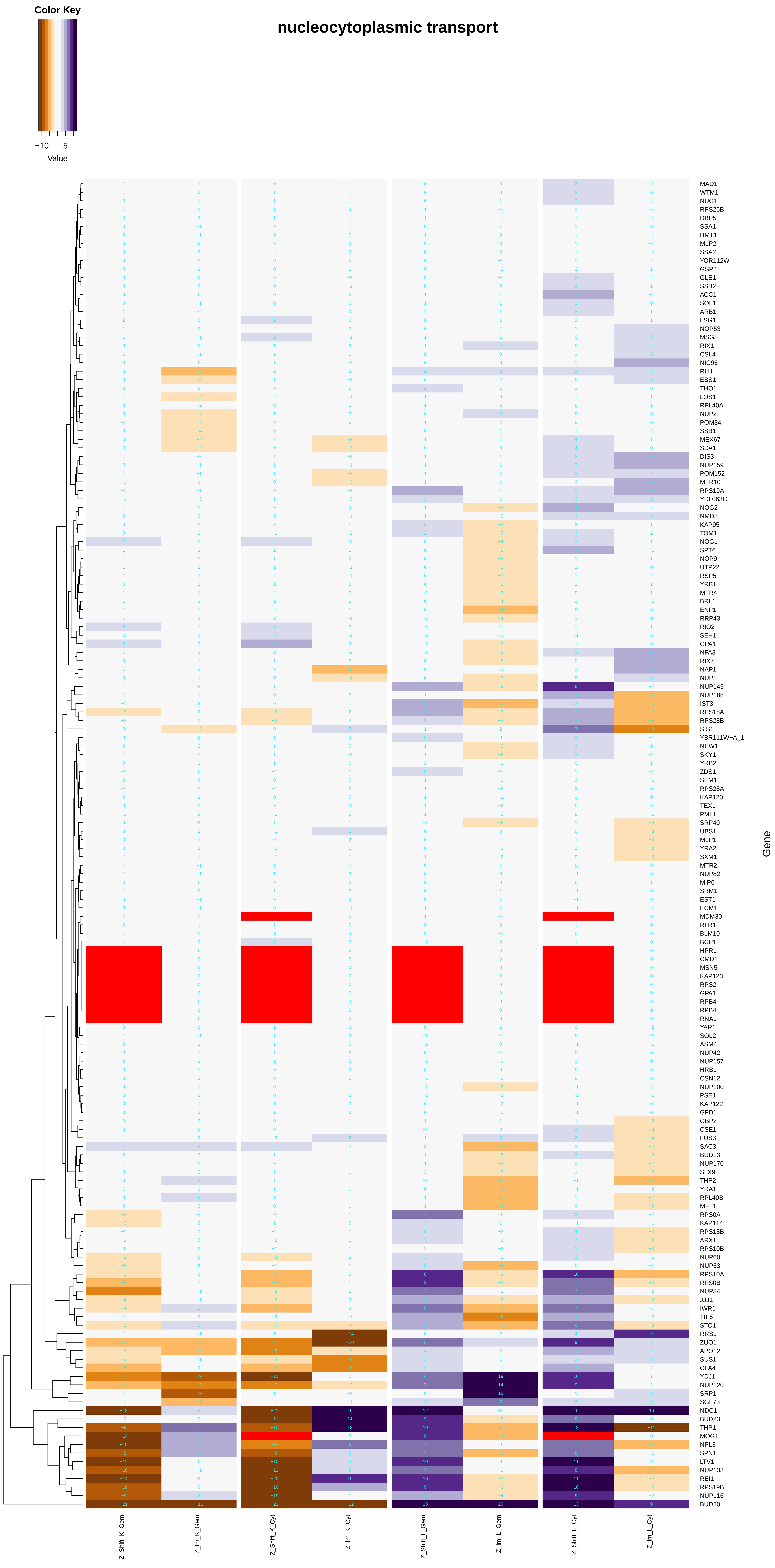
nucleocytoplasmic transport
Color Key
−10
5
Value
Gene
MAD1
WTM1
NUG1
RPS26B
DBP5
SSA1
HMT1
MLP2
SSA2
YOR112W
GSP2
GLE1
SSB2
ACC1
SOL1
ARB1
LSG1
NOP53
MSG5
RIX1
CSL4
NIC96
RLI1
EBS1
THO1
LOS1
RPL40A
NUP2
POM34
SSB1
MEX67
SDA1
DIS3
NUP159
POM152
MTR10
RPS19A
YDL063C
NOG2
NMD3
KAP95
TOM1
NOG1
SPT6
NOP9
UTP22
RSP5
YRB1
MTR4
BRL1
ENP1
RRP43
RIO2
SEH1
GPA1
NPA3
RIX7
NAP1
NUP1
NUP145
NUP188
IST3
RPS18A
RPS28B
SIS1
YBR111W−A_1
NEW1
SKY1
YRB2
ZDS1
SEM1
RPS28A
KAP120
TEX1
PML1
SRP40
UBS1
MLP1
YRA2
SXM1
MTR2
NUP82
MIP6
SRM1
EST1
ECM1
MDM30
RLR1
BLM10
BCP1
HPR1
CMD1
MSN5
KAP123
RPS2
GPA1
RPB4
RPB4
RNA1
YAR1
SOL2
ASM4
NUP42
NUP157
HRB1
CSN12
NUP100
PSE1
KAP122
GFD1
GBP2
CSE1
FUS3
SAC3
BUD13
NUP170
SLX9
THP2
YRA1
RPL40B
MFT1
RPS0A
KAP114
RPS18B
ARX1
RPS10B
NUP60
NUP53
RPS10A
RPS0B
NUP84
JJJ1
IWR1
TIF6
STO1
RRS1
ZUO1
APQ12
SUS1
CLA4
YDJ1
NUP120
SRP1
SGF73
NDC1
BUD23
THP1
MOG1
NPL3
SPN1
LTV1
NUP133
REI1
RPS19B
NUP116
BUD20
Z_Shift_K_Gem
Z_Im_K_Gem
Z_Shift_K_Cyt
Z_Im_K_Cyt
Z_Shift_L_Gem
Z_Im_L_Gem
Z_Shift_L_Cyt
Z_Im_L_Cyt

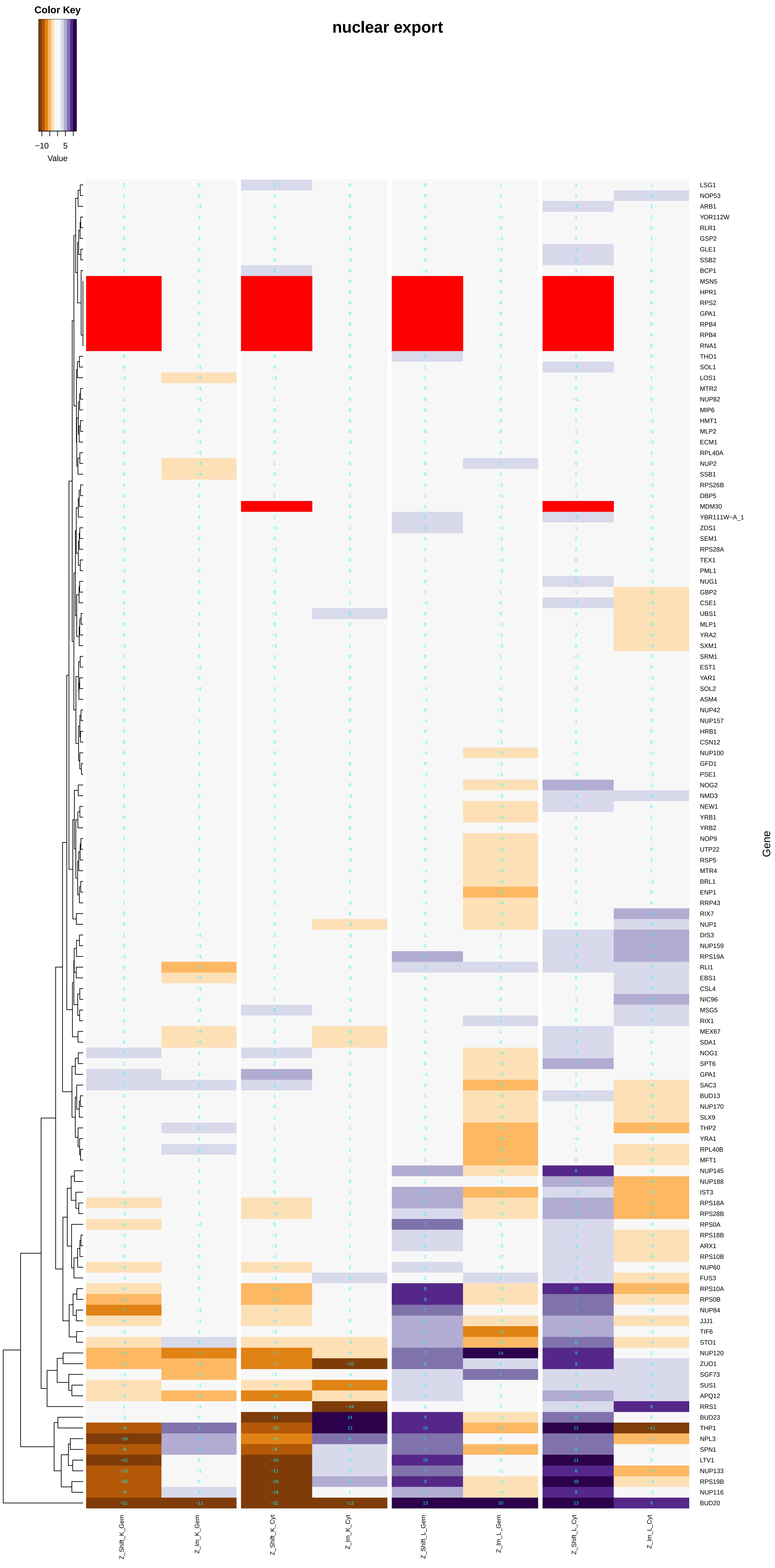
Color Key
−10
5
Value
nuclear export
LSG1
NOP53
ARB1
YOR112W
RLR1
GSP2
GLE1
SSB2
BCP1
MSN5
HPR1
RPS2
GPA1
RPB4
RPB4
RNA1
THO1
SOL1
LOS1
MTR2
NUP82
MIP6
HMT1
MLP2
ECM1
RPL40A
NUP2
SSB1
RPS26B
DBP5
MDM30
YBR111W−A_1
ZDS1
SEM1
RPS28A
TEX1
PML1
NUG1
GBP2
CSE1
UBS1
MLP1
YRA2
SXM1
SRM1
EST1
YAR1
SOL2
ASM4
NUP42
NUP157
HRB1
CSN12
NUP100
GFD1
PSE1
NOG2
NMD3
NEW1
YRB1
YRB2
NOP9
UTP22
RSP5
MTR4
BRL1
ENP1
RRP43
RIX7
NUP1
DIS3
NUP159
RPS19A
RLI1
EBS1
CSL4
NIC96
MSG5
RIX1
MEX67
SDA1
NOG1
SPT6
GPA1
SAC3
BUD13
NUP170
SLX9
THP2
YRA1
RPL40B
MFT1
NUP145
NUP188
IST3
RPS18A
RPS28B
RPS0A
RPS18B
ARX1
RPS10B
NUP60
FUS3
RPS10A
RPS0B
NUP84
JJJ1
TIF6
STO1
NUP120
ZUO1
SGF73
SUS1
APQ12
RRS1
BUD23
THP1
NPL3
SPN1
LTV1
NUP133
RPS19B
NUP116
BUD20
Gene
Z_Shift_K_Gem
Z_Im_K_Gem
Z_Shift_K_Cyt
Z_Im_K_Cyt
Z_Shift_L_Gem
Z_Im_L_Gem
Z_Shift_L_Cyt
Z_Im_L_Cyt

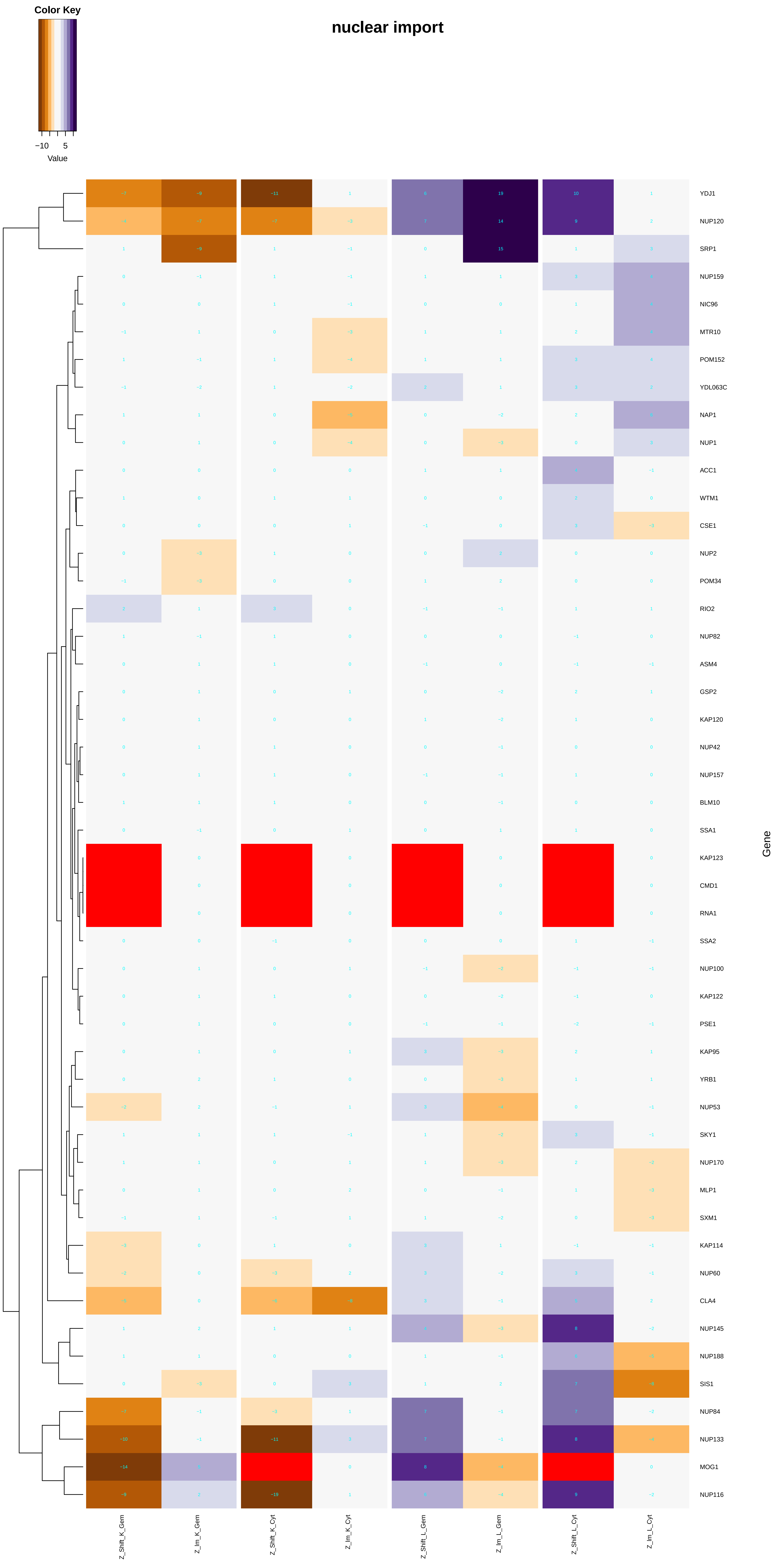
Color Key
−10
5
Value
nuclear import
YDJ1
NUP120
SRP1
NUP159
NIC96
MTR10
POM152
YDL063C
NAP1
NUP1
ACC1
WTM1
CSE1
NUP2
POM34
RIO2
NUP82
ASM4
GSP2
KAP120
NUP42
NUP157
BLM10
SSA1
KAP123
CMD1
RNA1
SSA2
NUP100
KAP122
PSE1
KAP95
YRB1
NUP53
SKY1
NUP170
MLP1
SXM1
KAP114
NUP60
CLA4
NUP145
NUP188
SIS1
NUP84
NUP133
MOG1
NUP116
Gene
Z_Shift_K_Gem
Z_Im_K_Gem
Z_Shift_K_Cyt
Z_Im_K_Cyt
Z_Shift_L_Gem
Z_Im_L_Gem
Z_Shift_L_Cyt
Z_Im_L_Cyt

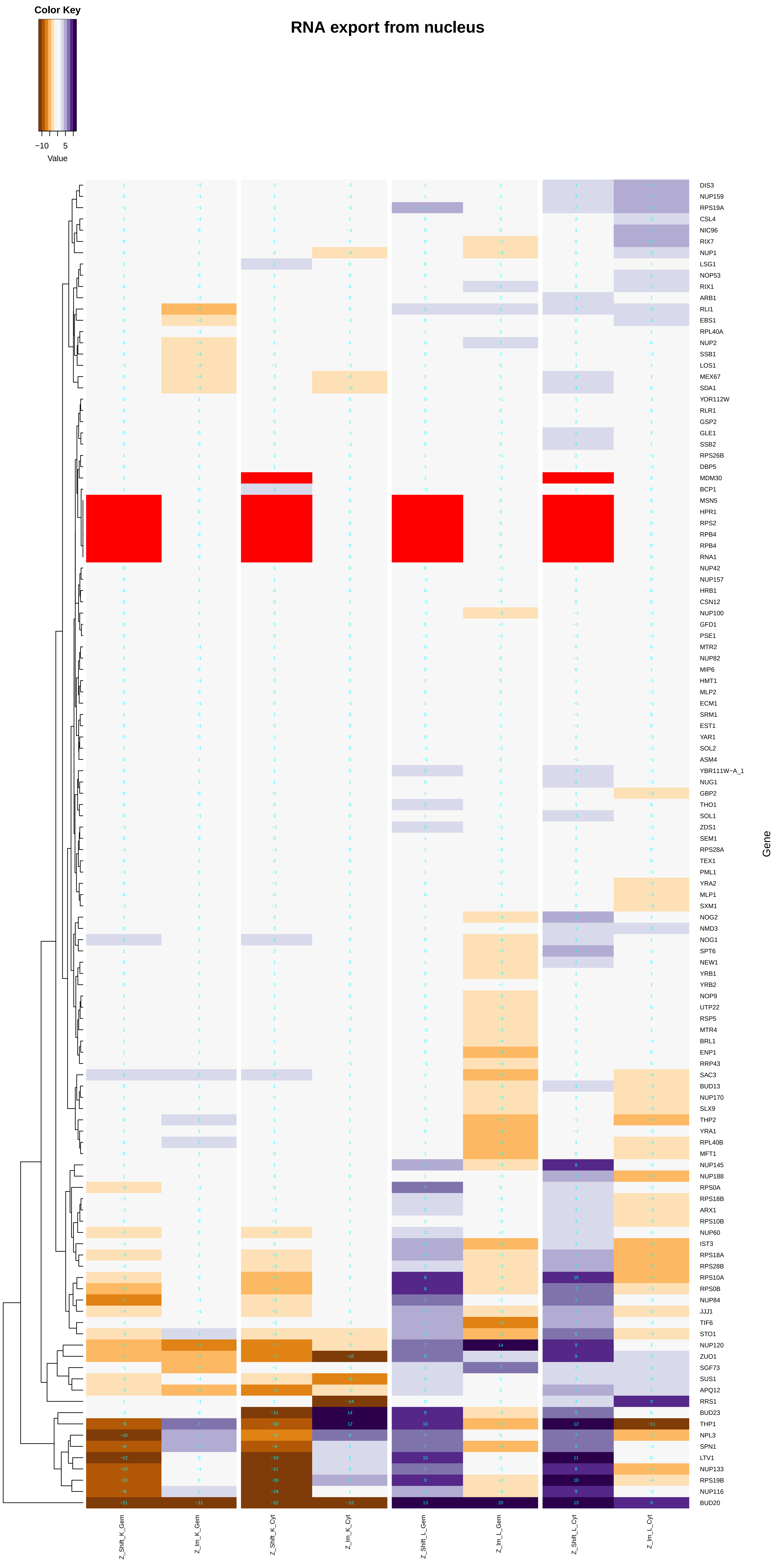
Color Key
−10
5
Value
RNA export from nucleus
DIS3
NUP159
RPS19A
CSL4
NIC96
RIX7
NUP1
LSG1
NOP53
RIX1
ARB1
RLI1
EBS1
RPL40A
NUP2
SSB1
LOS1
MEX67
SDA1
YOR112W
RLR1
GSP2
GLE1
SSB2
RPS26B
DBP5
MDM30
BCP1
MSN5
HPR1
RPS2
RPB4
RPB4
RNA1
NUP42
NUP157
HRB1
CSN12
NUP100
GFD1
PSE1
MTR2
NUP82
MIP6
HMT1
MLP2
ECM1
SRM1
EST1
YAR1
SOL2
ASM4
YBR111W−A_1
NUG1
GBP2
THO1
SOL1
ZDS1
SEM1
RPS28A
TEX1
PML1
YRA2
MLP1
SXM1
NOG2
NMD3
NOG1
SPT6
NEW1
YRB1
YRB2
NOP9
UTP22
RSP5
MTR4
BRL1
ENP1
RRP43
SAC3
BUD13
NUP170
SLX9
THP2
YRA1
RPL40B
MFT1
NUP145
NUP188
RPS0A
RPS18B
ARX1
RPS10B
NUP60
IST3
RPS18A
RPS28B
RPS10A
RPS0B
NUP84
JJJ1
TIF6
STO1
NUP120
ZUO1
SGF73
SUS1
APQ12
RRS1
BUD23
THP1
NPL3
SPN1
LTV1
NUP133
RPS19B
NUP116
BUD20
Gene
Z_Shift_K_Gem
Z_Im_K_Gem
Z_Shift_K_Cyt
Z_Im_K_Cyt
Z_Shift_L_Gem
Z_Im_L_Gem
Z_Shift_L_Cyt
Z_Im_L_Cyt

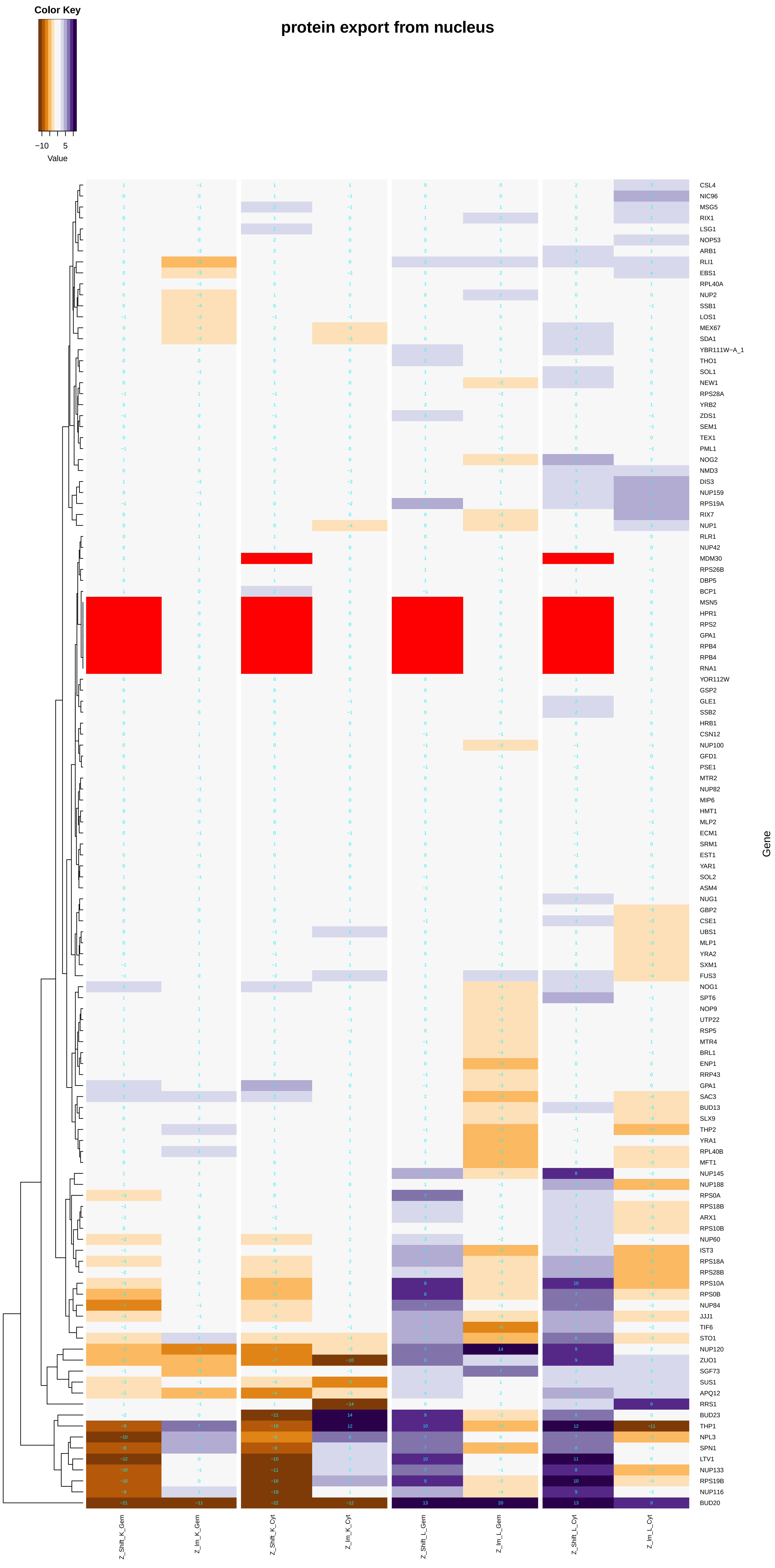
Color Key
−10
5
Value
protein export from nucleus
CSL4
NIC96
MSG5
RIX1
LSG1
NOP53
ARB1
RLI1
EBS1
RPL40A
NUP2
SSB1
LOS1
MEX67
SDA1
YBR111W−A_1
THO1
SOL1
NEW1
RPS28A
YRB2
ZDS1
SEM1
TEX1
PML1
NOG2
NMD3
DIS3
NUP159
RPS19A
RIX7
NUP1
RLR1
NUP42
MDM30
RPS26B
DBP5
BCP1
MSN5
HPR1
RPS2
GPA1
RPB4
RPB4
RNA1
YOR112W
GSP2
GLE1
SSB2
HRB1
CSN12
NUP100
GFD1
PSE1
MTR2
NUP82
MIP6
HMT1
MLP2
ECM1
SRM1
EST1
YAR1
SOL2
ASM4
NUG1
GBP2
CSE1
UBS1
MLP1
YRA2
SXM1
FUS3
NOG1
SPT6
NOP9
UTP22
RSP5
MTR4
BRL1
ENP1
RRP43
GPA1
SAC3
BUD13
SLX9
THP2
YRA1
RPL40B
MFT1
NUP145
NUP188
RPS0A
RPS18B
ARX1
RPS10B
NUP60
IST3
RPS18A
RPS28B
RPS10A
RPS0B
NUP84
JJJ1
TIF6
STO1
NUP120
ZUO1
SGF73
SUS1
APQ12
RRS1
BUD23
THP1
NPL3
SPN1
LTV1
NUP133
RPS19B
NUP116
BUD20
Gene
Z_Shift_K_Gem
Z_lm_K_Gem
Z_Shift_K_Cyt
Z_lm_K_Cyt
Z_Shift_L_Gem
Z_lm_L_Gem
Z_Shift_L_Cyt
Z_lm_L_Cyt

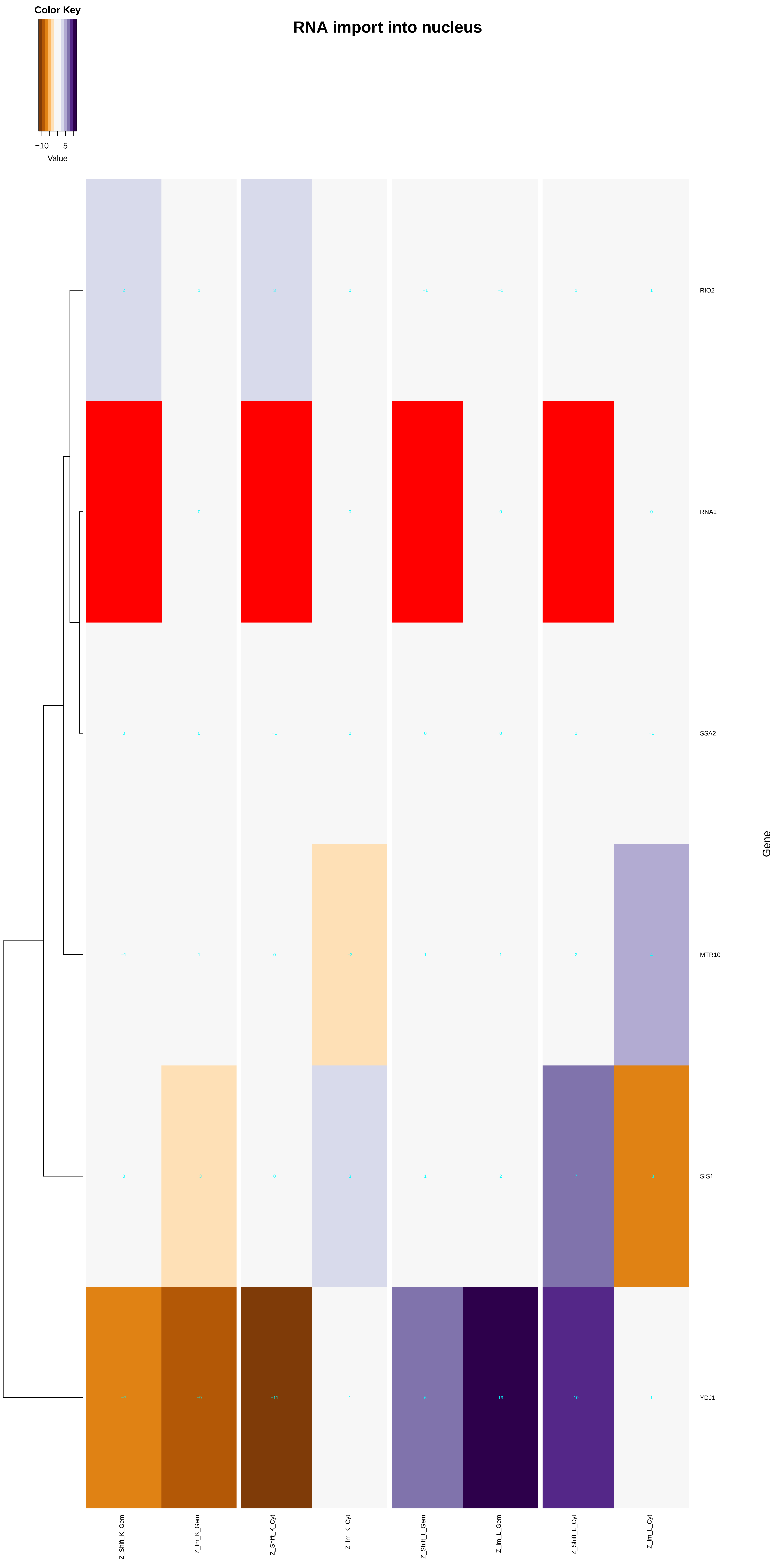

Color Key
−10
5
Value
RNA import into nucleus
RIO2
RNA1
SSA2
MTR10
SIS1
YDJ1
Gene
Z_Shift_K_Gem
Z_Im_K_Gem
Z_Shift_K_Cyt
Z_Im_K_Cyt
Z_Shift_L_Gem
Z_Im_L_Gem
Z_Shift_L_Cyt
Z_Im_L_Cyt

mRNA export from nucleus

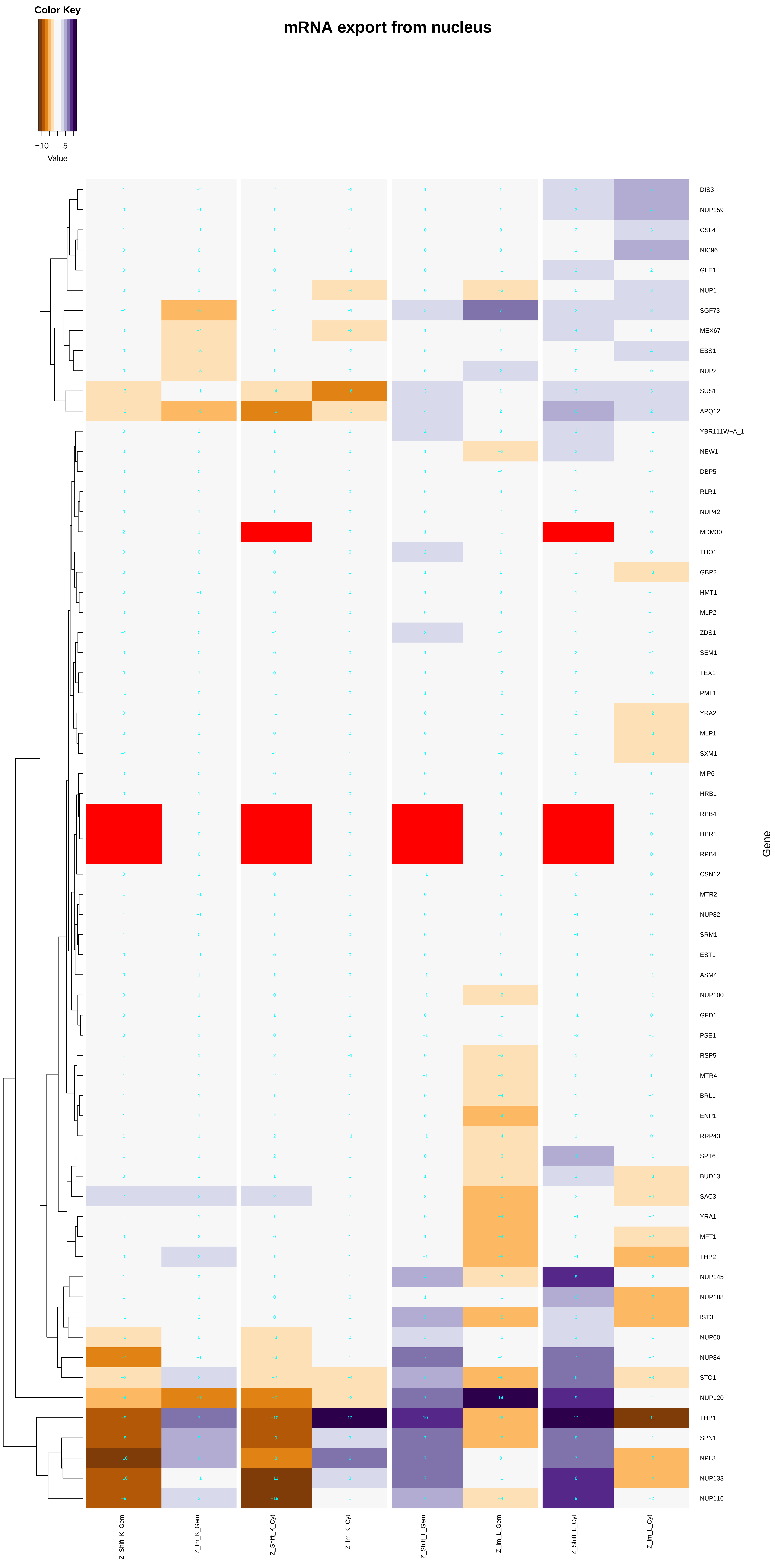

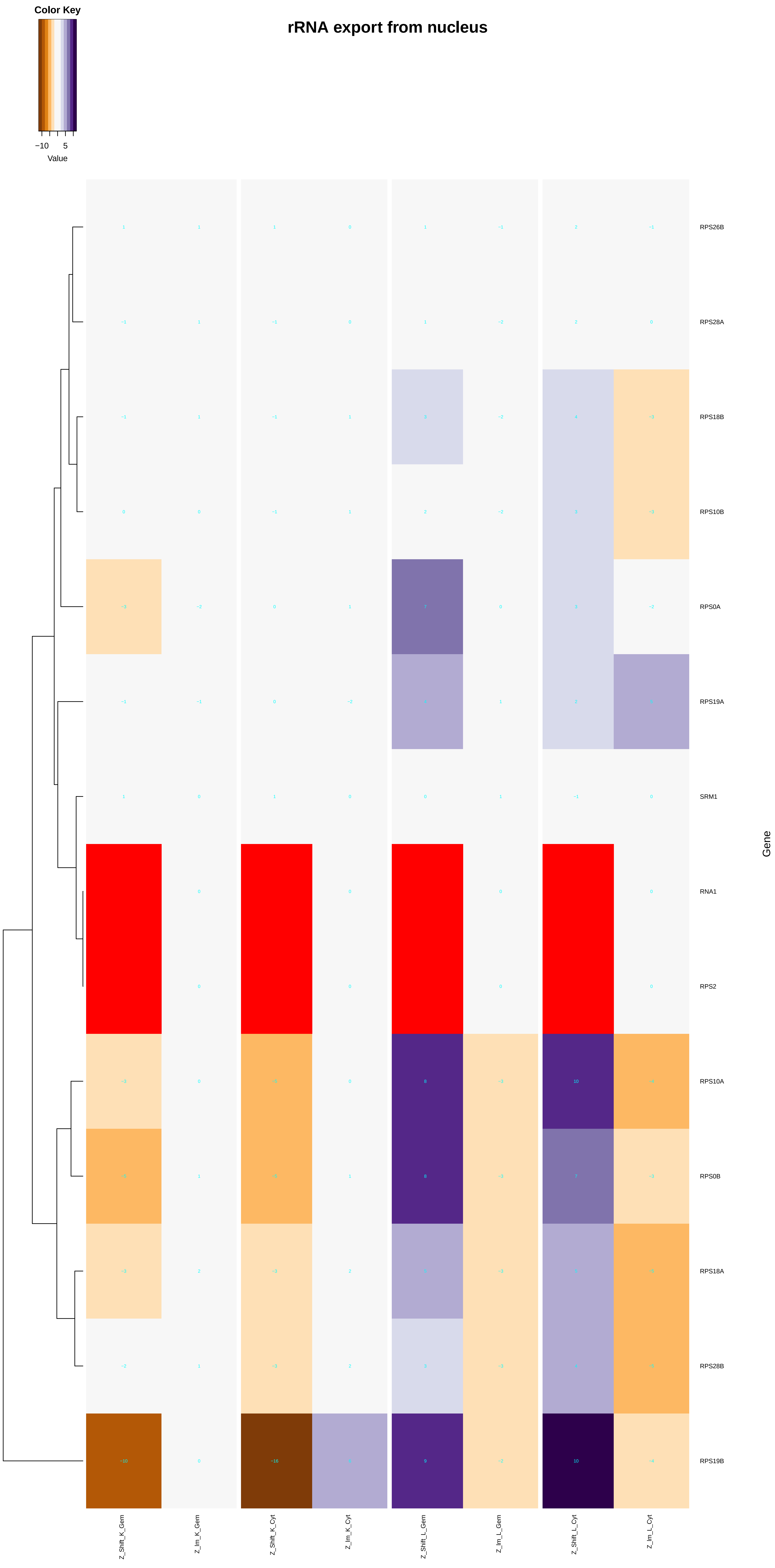
Color Key
−10
5
Value
rRNA export from nucleus
RPS26B
RPS28A
RPS18B
RPS10B
RPS0A
RPS19A
SRM1
RNA1
RPS2
RPS10A
RPS0B
RPS18A
RPS28B
RPS19B
Gene
Z_Shift_K_Gem
Z_Im_K_Gem
Z_Shift_K_Cyt
Z_Im_K_Cyt
Z_Shift_L_Gem
Z_Im_L_Gem
Z_Shift_L_Cyt
Z_Im_L_Cyt

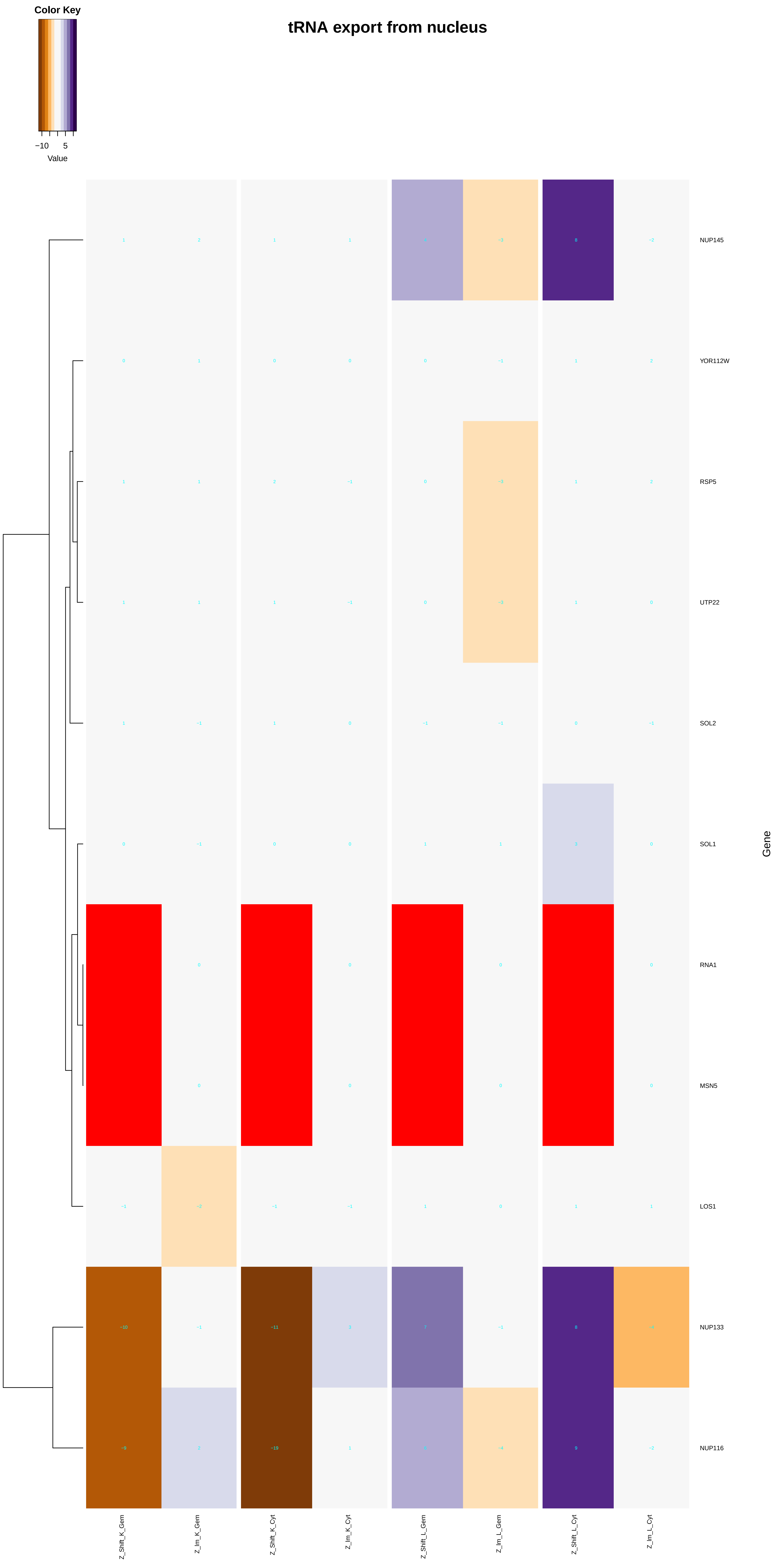
Color Key
−10
5
Value
tRNA export from nucleus
NUP145
YOR112W
RSP5
UTP22
SOL2
SOL1
RNA1
MSN5
LOS1
NUP133
NUP116
Gene
Z_Shift_K_Gem
Z_Im_K_Gem
Z_Shift_K_Cyt
Z_Im_K_Cyt
Z_Shift_L_Gem
Z_Im_L_Gem
Z_Shift_L_Cyt
Z_Im_L_Cyt

ribonucleoprotein complex export from nucleus

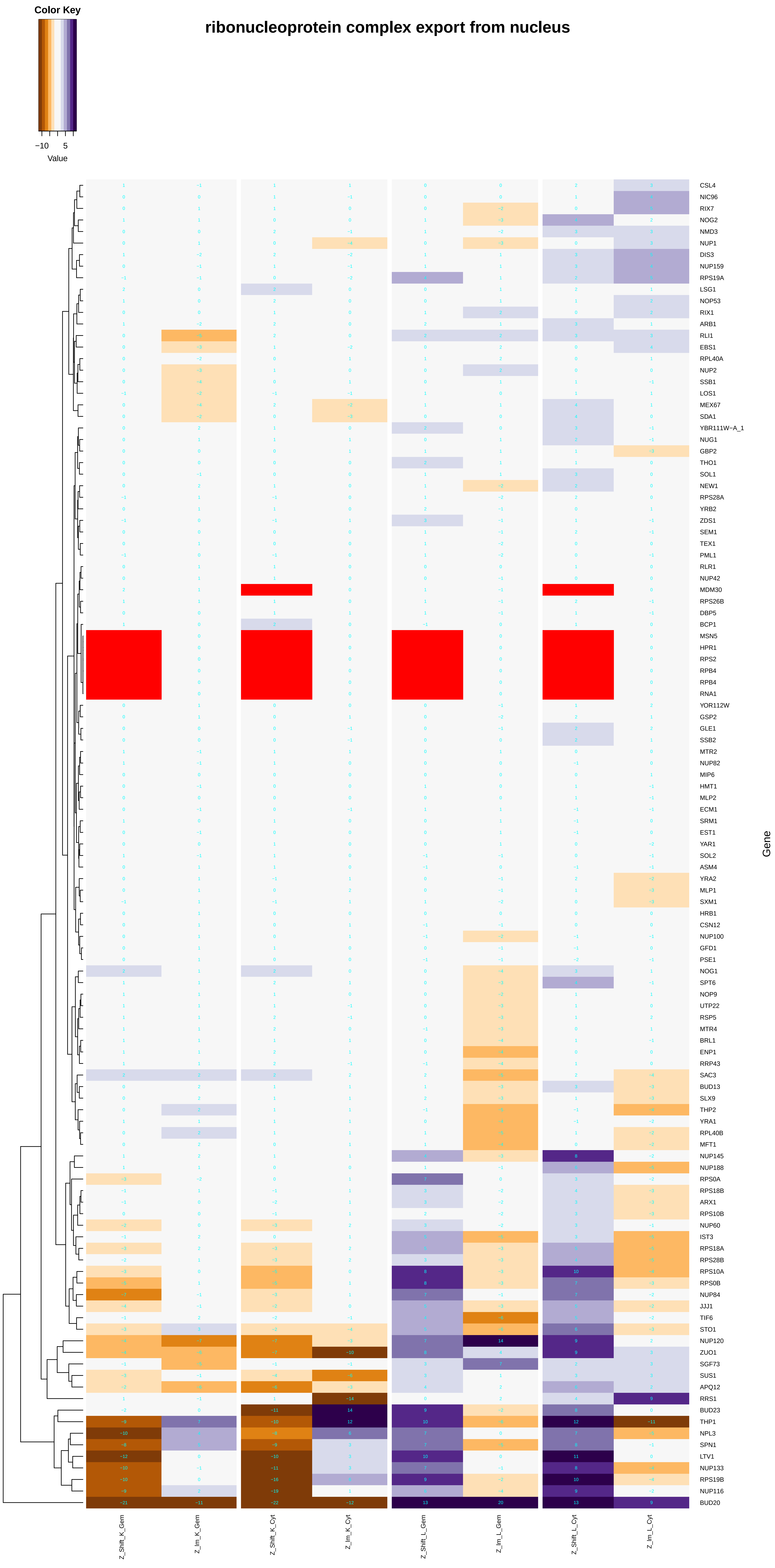

ncRNA export from nucleus

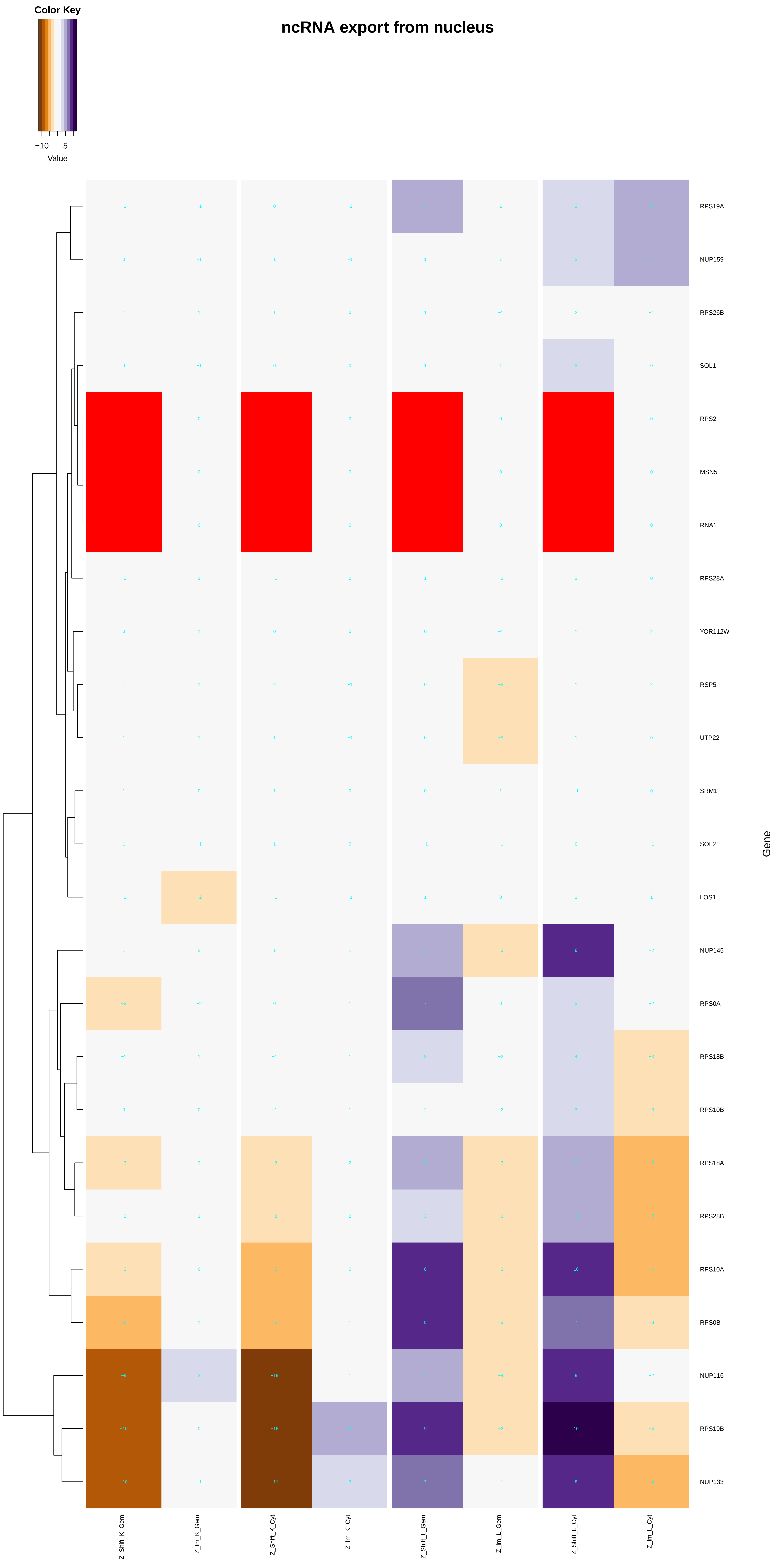

MAPK export from nucleus

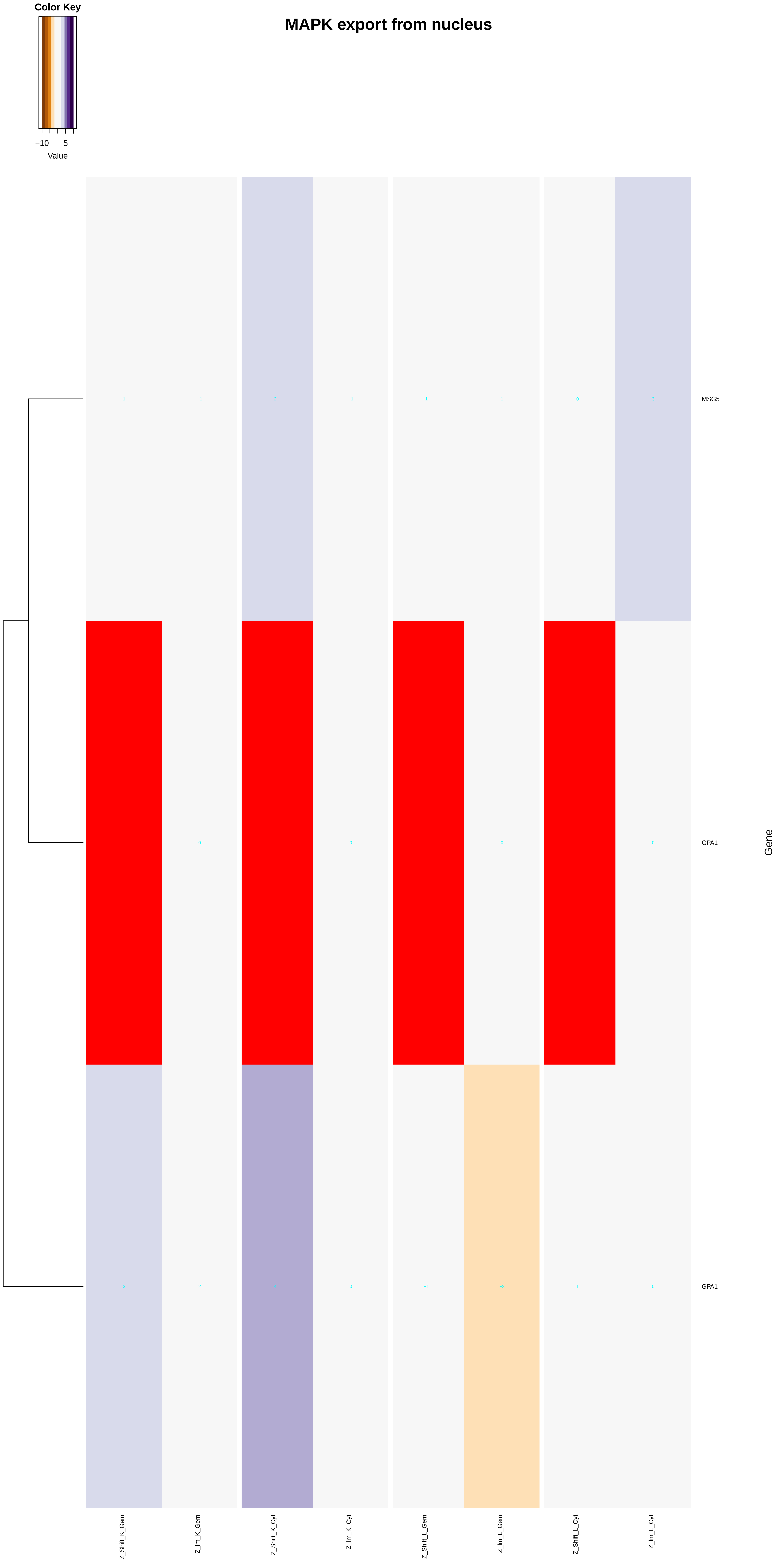

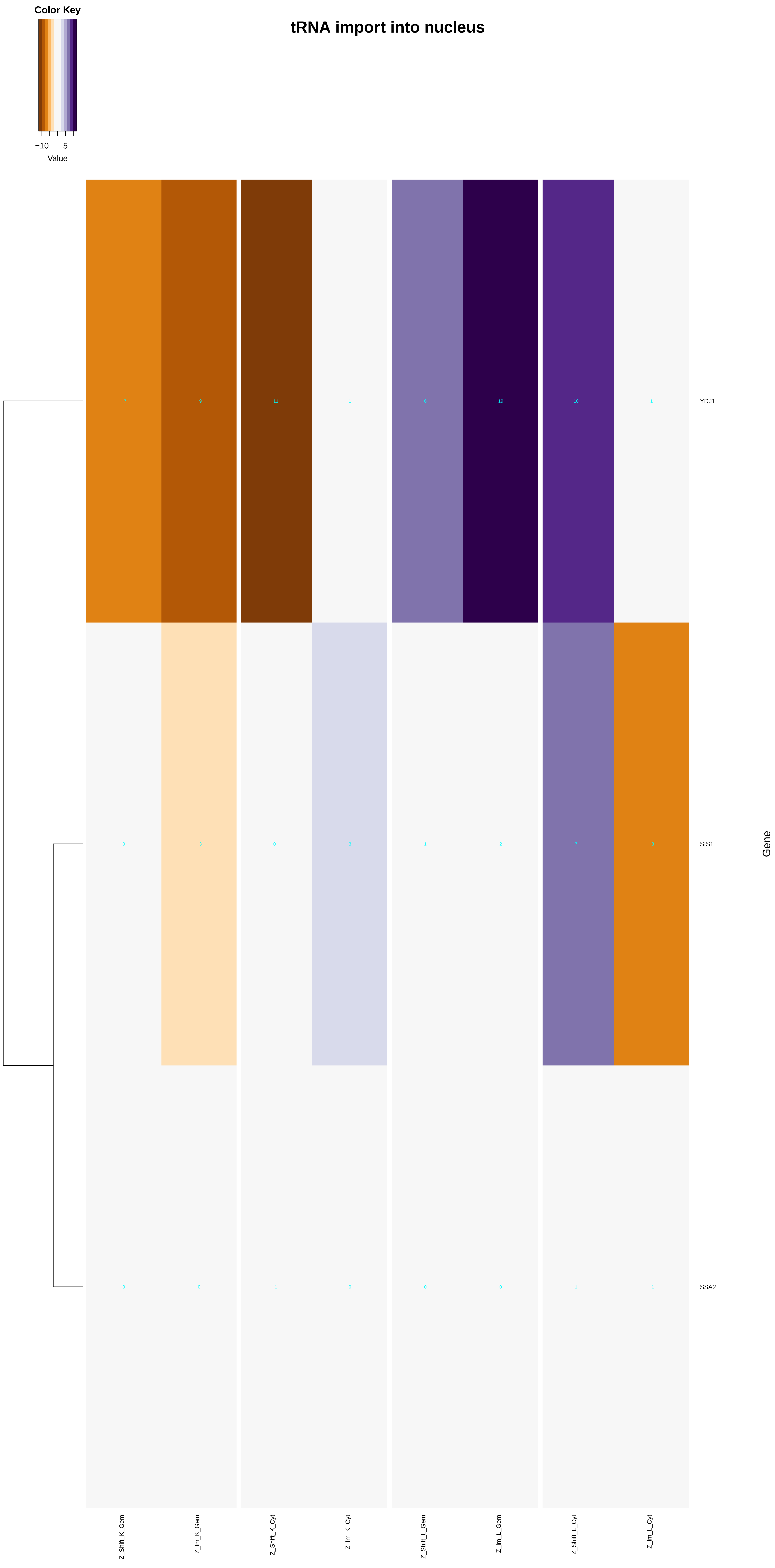
Color Key
−10
5
Value
tRNA import into nucleus
−7
−9
−11
1
6
19
10
1
YDJ1
0
−3
0
3
1
2
7
−8
SIS1
0
0
−1
0
0
0
1
−1
SSA2
Gene
Z_Shift_K_Gem
Z_Im_K_Gem
Z_Shift_K_Cyt
Z_Im_K_Cyt
Z_Shift_L_Gem
Z_Im_L_Gem
Z_Shift_L_Cyt
Z_Im_L_Cyt

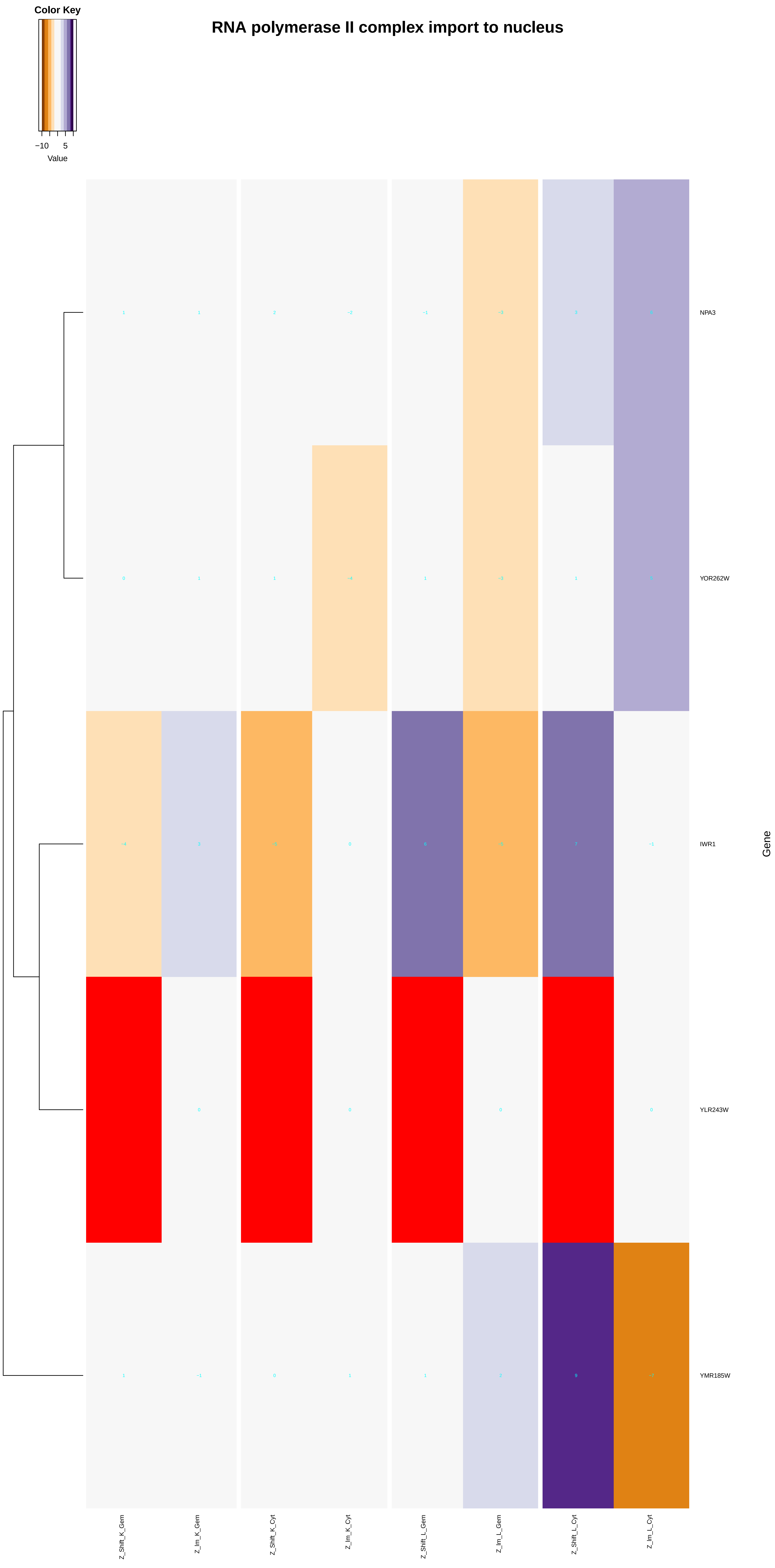
Color Key
−10
5
Value
RNA polymerase II complex import to nucleus
NPA3
YOR262W
IWR1
YLR243W
YMR185W
Gene
Z_Shift_K_Gem
Z_Im_K_Gem
Z_Shift_K_Cyt
Z_Im_K_Cyt
Z_Shift_L_Gem
Z_Im_L_Gem
Z_Shift_L_Cyt
Z_Im_L_Cyt

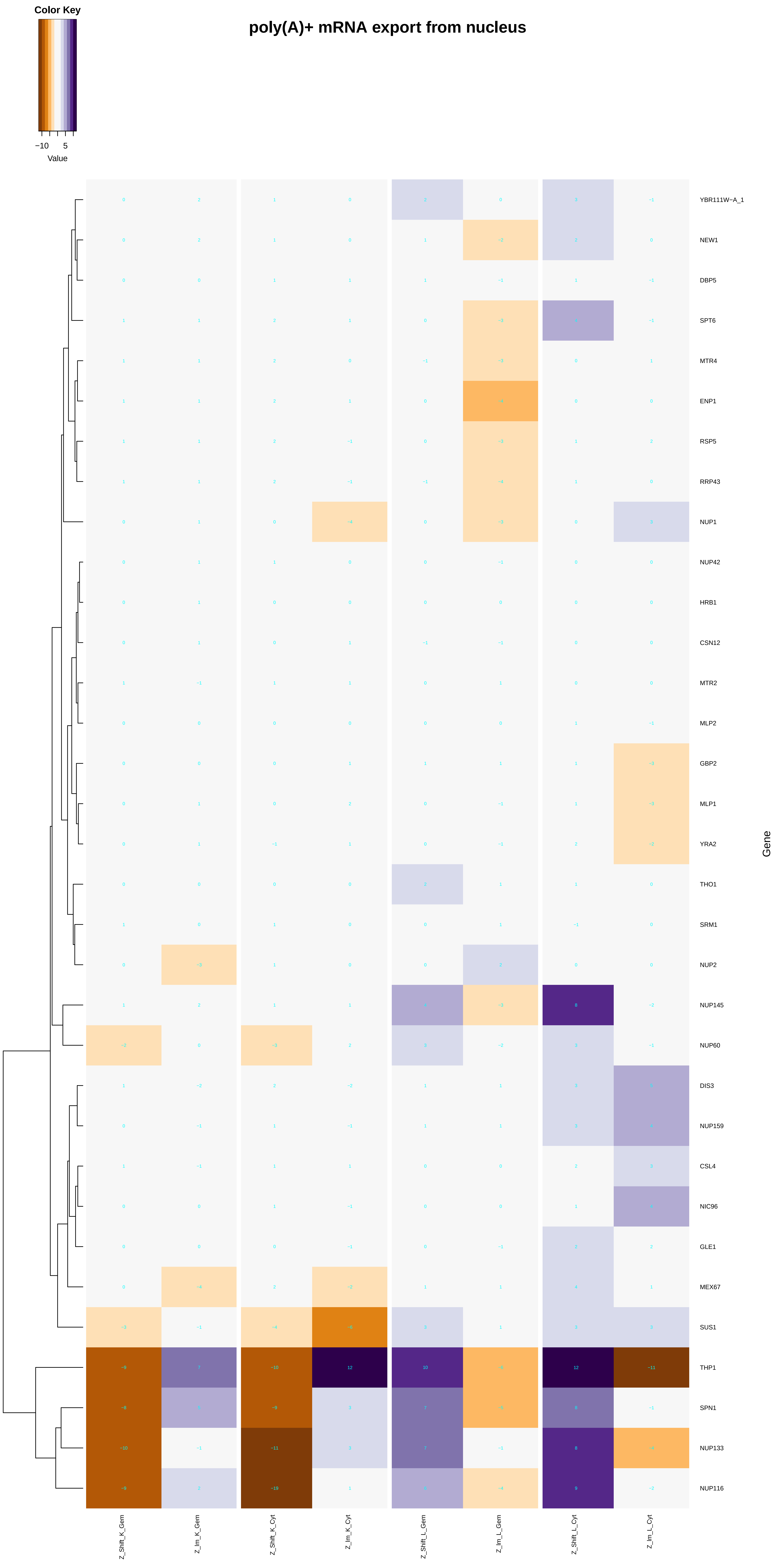

poly(A)+ mRNA export from nucleus
Color Key
−10
5
Value
YBR111W−A_1
NEW1
DBP5
SPT6
MTR4
ENP1
RSP5
RRP43
NUP1
NUP42
HRB1
CSN12
MTR2
MLP2
GBP2
MLP1
YRA2
THO1
SRM1
NUP2
NUP145
NUP60
DIS3
NUP159
CSL4
NIC96
GLE1
MEX67
SUS1
THP1
SPN1
NUP133
NUP116
Gene
Z_Shift_K_Gem
Z_Im_K_Gem
Z_Shift_K_Cyt
Z_Im_K_Cyt
Z_Shift_L_Gem
Z_Im_L_Gem
Z_Shift_L_Cyt
Z_Im_L_Cyt

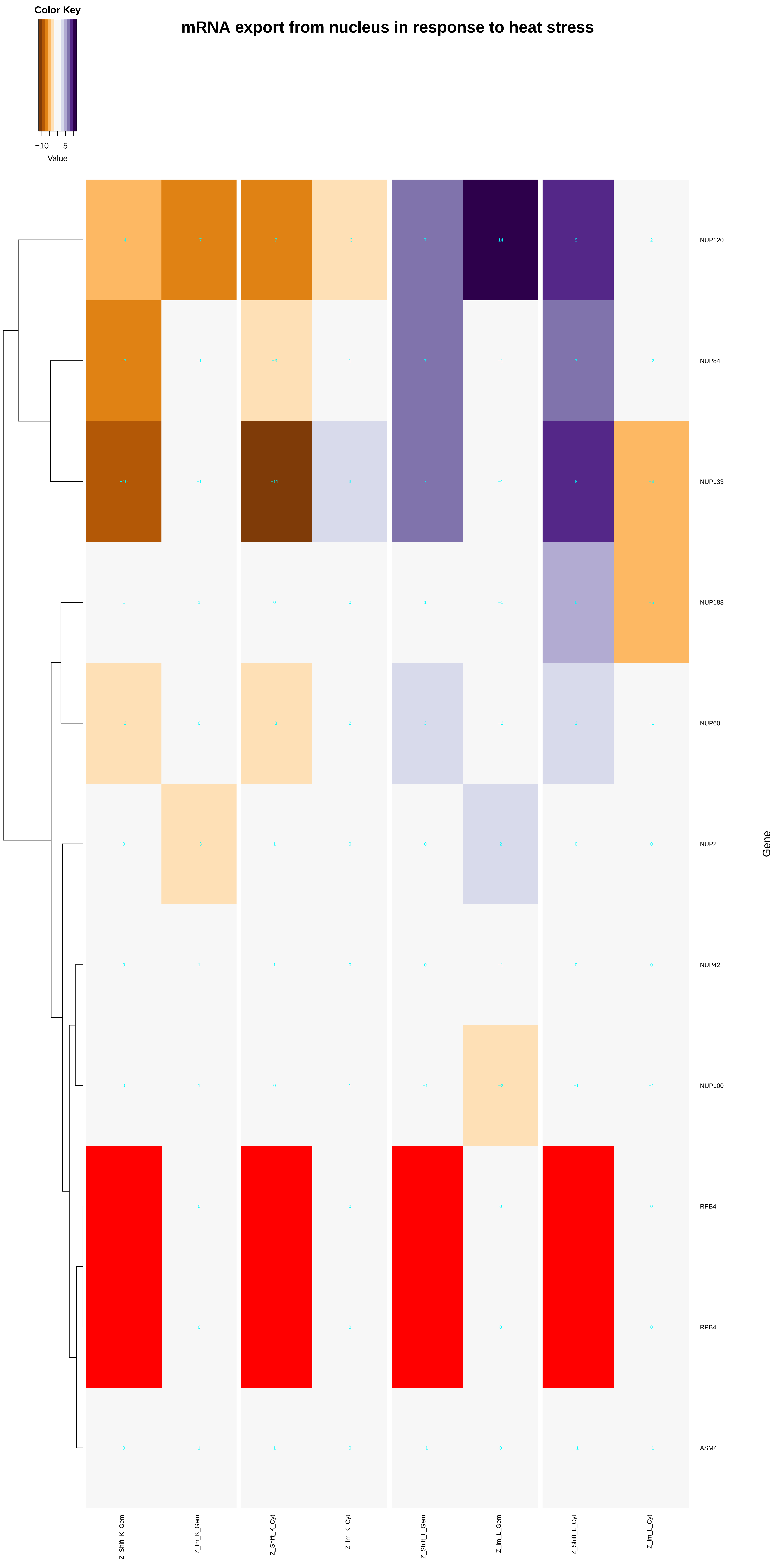

Color Key
−10
5
Value
mRNA export from nucleus in response to heat stress
NUP120
NUP84
NUP133
NUP188
NUP60
NUP2
NUP42
NUP100
RPB4
RPB4
ASM4
Gene
Z_Shift_K_Gem
Z_Im_K_Gem
Z_Shift_K_Cyt
Z_Im_K_Cyt
Z_Shift_L_Gem
Z_Im_L_Gem
Z_Shift_L_Cyt
Z_Im_L_Cyt

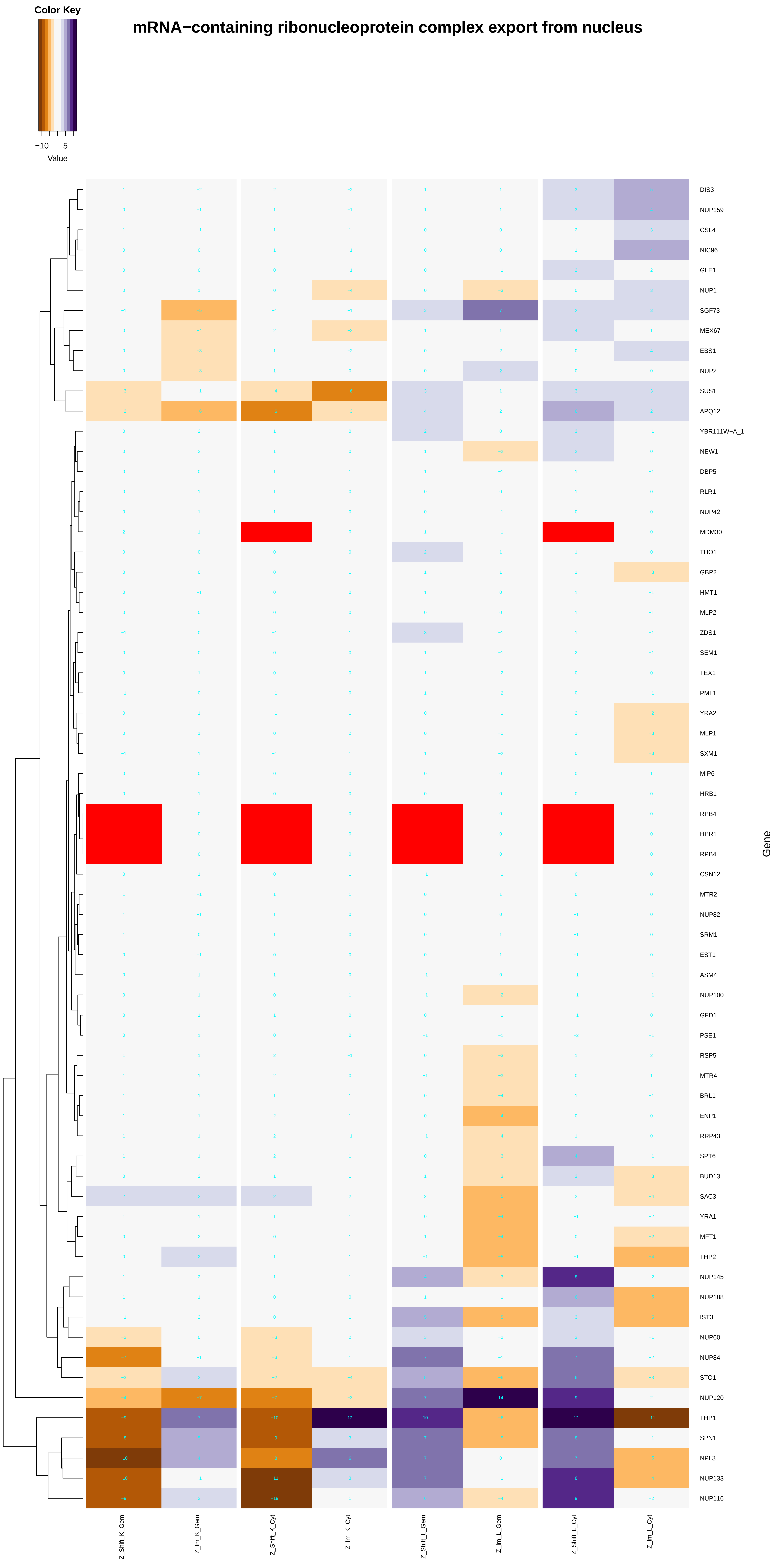

Color Key
−10
5
Value
mRNA−containing ribonucleoprotein complex export from nucleus
DIS3
NUP159
CSL4
NIC96
GLE1
NUP1
SGF73
MEX67
EBS1
NUP2
SUS1
APQ12
YBR111W−A_1
NEW1
DBP5
RLR1
NUP42
MDM30
THO1
GBP2
HMT1
MLP2
ZDS1
SEM1
TEX1
PML1
YRA2
MLP1
SXM1
MIP6
HRB1
RPB4
HPR1
RPB4
CSN12
MTR2
NUP82
SRM1
EST1
ASM4
NUP100
GFD1
PSE1
RSP5
MTR4
BRL1
ENP1
RRP43
SPT6
BUD13
SAC3
YRA1
MFT1
THP2
NUP145
NUP188
IST3
NUP60
NUP84
STO1
NUP120
THP1
SPN1
NPL3
NUP133
NUP116
Gene
Z_Shift_K_Gem
Z_lm_K_Gem
Z_Shift_K_Cyt
Z_lm_K_Cyt
Z_Shift_L_Gem
Z_lm_L_Gem
Z_Shift_L_Cyt
Z_lm_L_Cyt

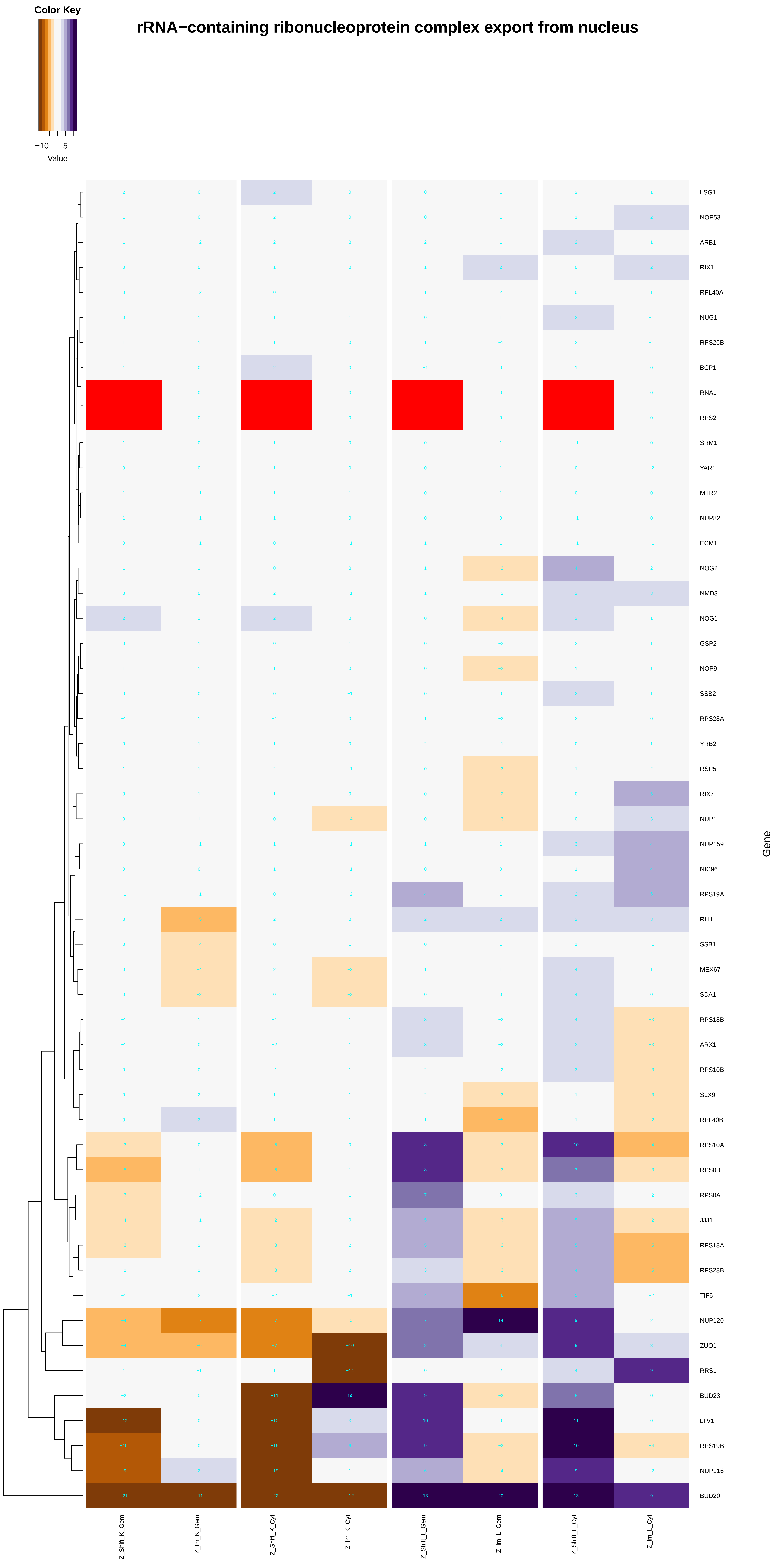
Color Key
−10 5
Value
rRNA−containing ribonucleoprotein complex export from nucleus
LSG1
NOP53
ARB1
RIX1
RPL40A
NUG1
RPS26B
BCP1
RNA1
RPS2
SRM1
YAR1
MTR2
NUP82
ECM1
NOG2
NMD3
NOG1
GSP2
NOP9
SSB2
RPS28A
YRB2
RSP5
RIX7
NUP1
NUP159
NIC96
RPS19A
RLI1
SSB1
MEX67
SDA1
RPS18B
ARX1
RPS10B
SLX9
RPL40B
RPS10A
RPS0B
RPS0A
JJJ1
RPS18A
RPS28B
TIF6
NUP120
ZUO1
RRS1
BUD23
LTV1
RPS19B
NUP116
BUD20
Gene
Z_Shift_K_Gem
Z_Im_K_Gem
Z_Shift_K_Cyt
Z_Im_K_Cyt
Z_Shift_L_Gem
Z_Im_L_Gem
Z_Shift_L_Cyt
Z_Im_L_Cyt

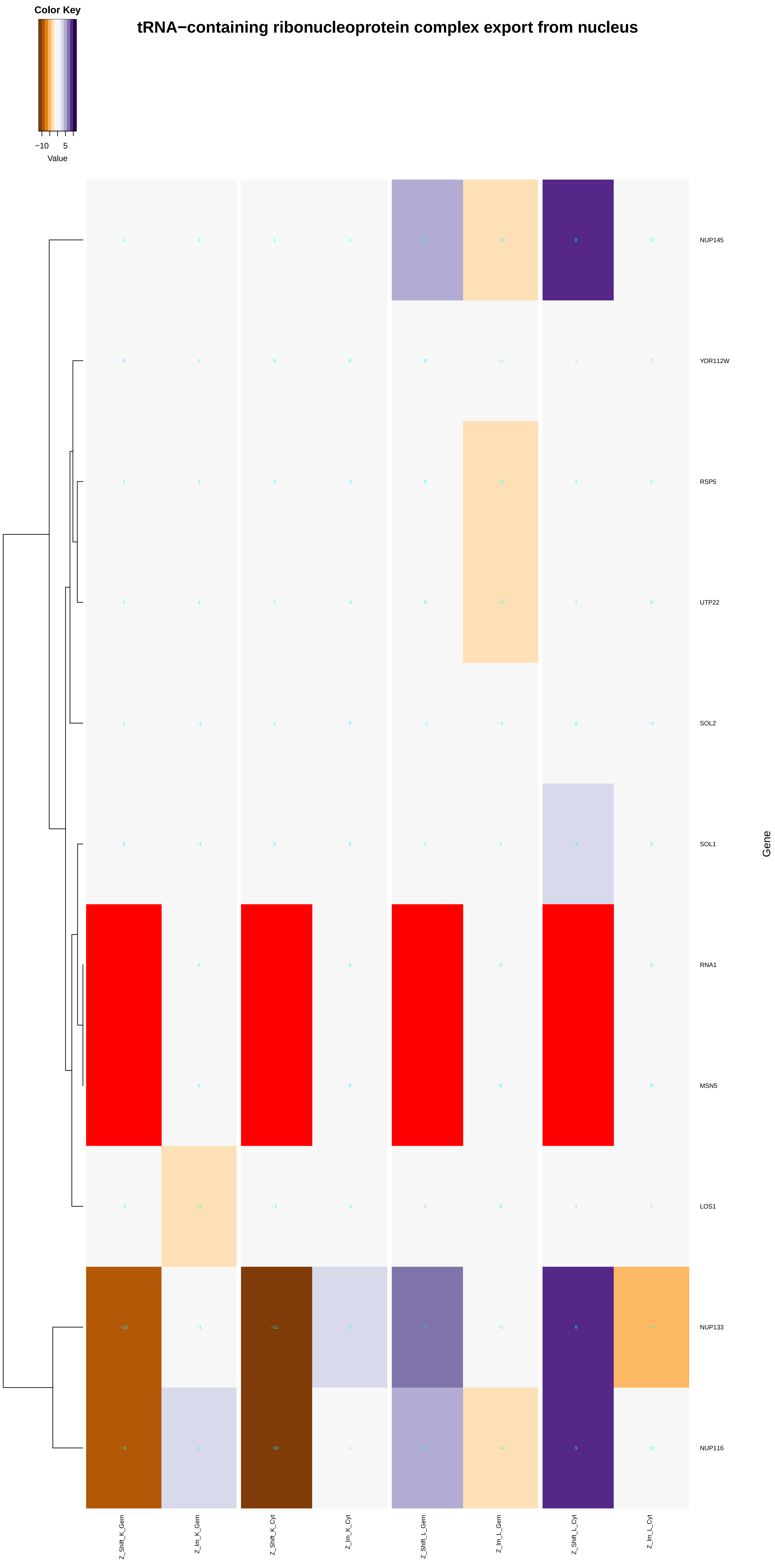

Color Key
−10
5
Value
tRNA−containing ribonucleoprotein complex export from nucleus
NUP145
YOR112W
RSP5
UTP22
SOL2
SOL1
RNA1
MSN5
LOS1
NUP133
NUP116
Gene
Z_Shift_K_Gem
Z_Im_K_Gem
Z_Shift_K_Cyt
Z_Im_K_Cyt
Z_Shift_L_Gem
Z_Im_L_Gem
Z_Shift_L_Cyt
Z_Im_L_Cyt

ribosomal subunit export from nucleus

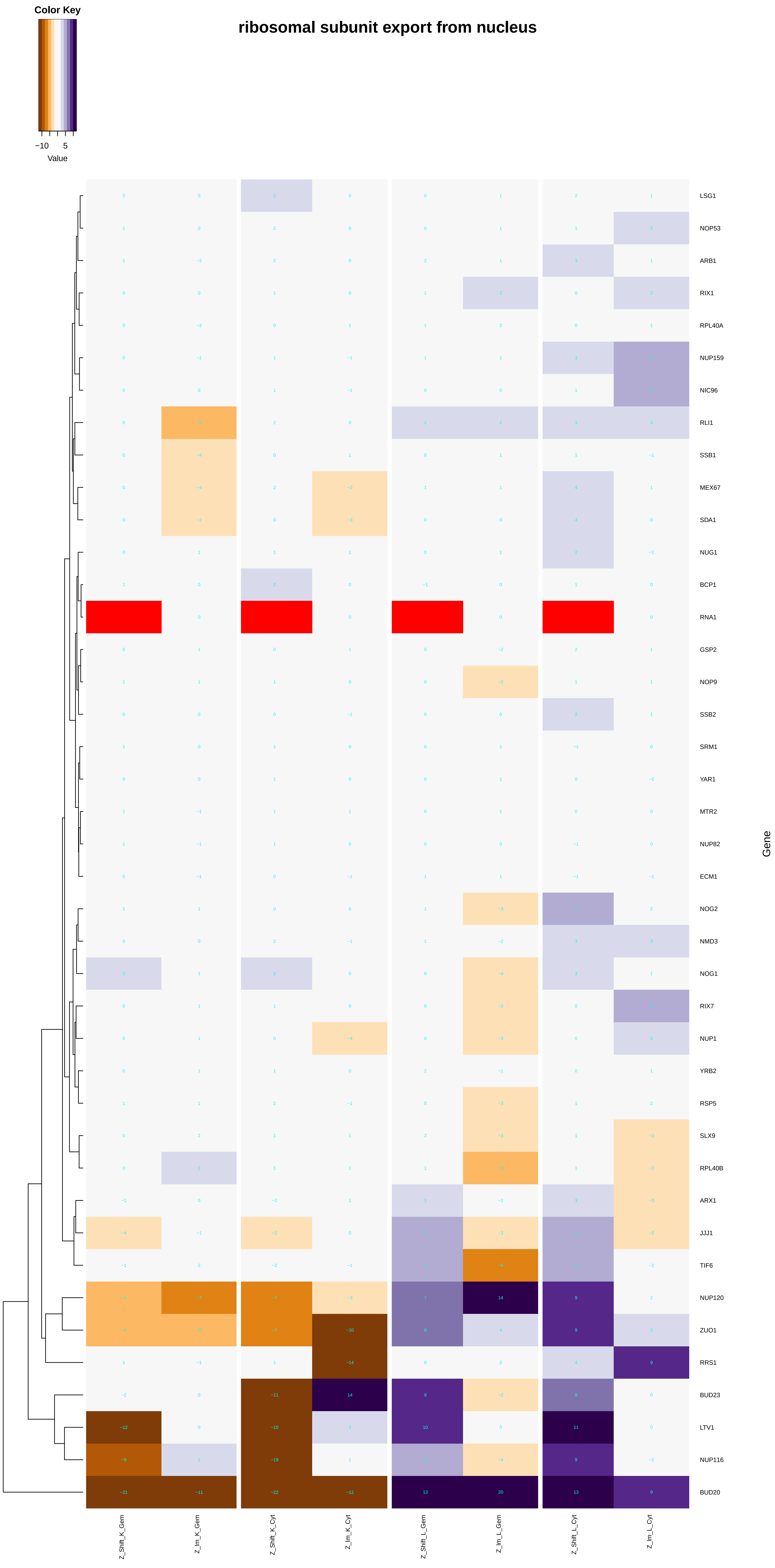

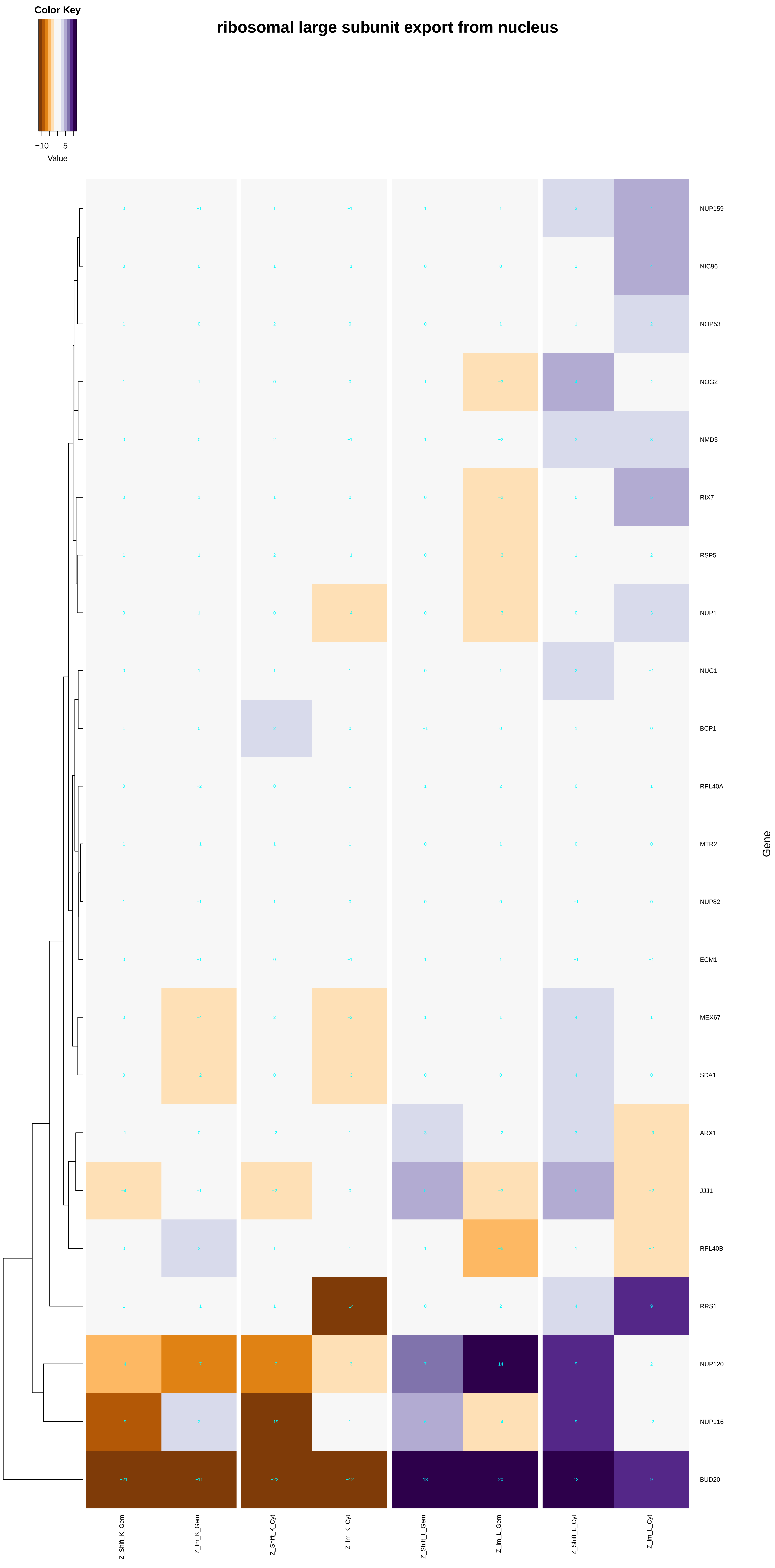
Color Key
−10 5
Value
ribosomal large subunit export from nucleus
NUP159
NIC96
NOP53
NOG2
NMD3
RIX7
RSP5
NUP1
NUG1
BCP1
RPL40A
MTR2
NUP82
ECM1
MEX67
SDA1
ARX1
JJJ1
RPL40B
RRS1
NUP120
NUP116
BUD20
Gene
Z_Shift_K_Gem
Z_lm_K_Gem
Z_Shift_K_Cyt
Z_lm_K_Cyt
Z_Shift_L_Gem
Z_lm_L_Gem
Z_Shift_L_Cyt
Z_lm_L_Cyt

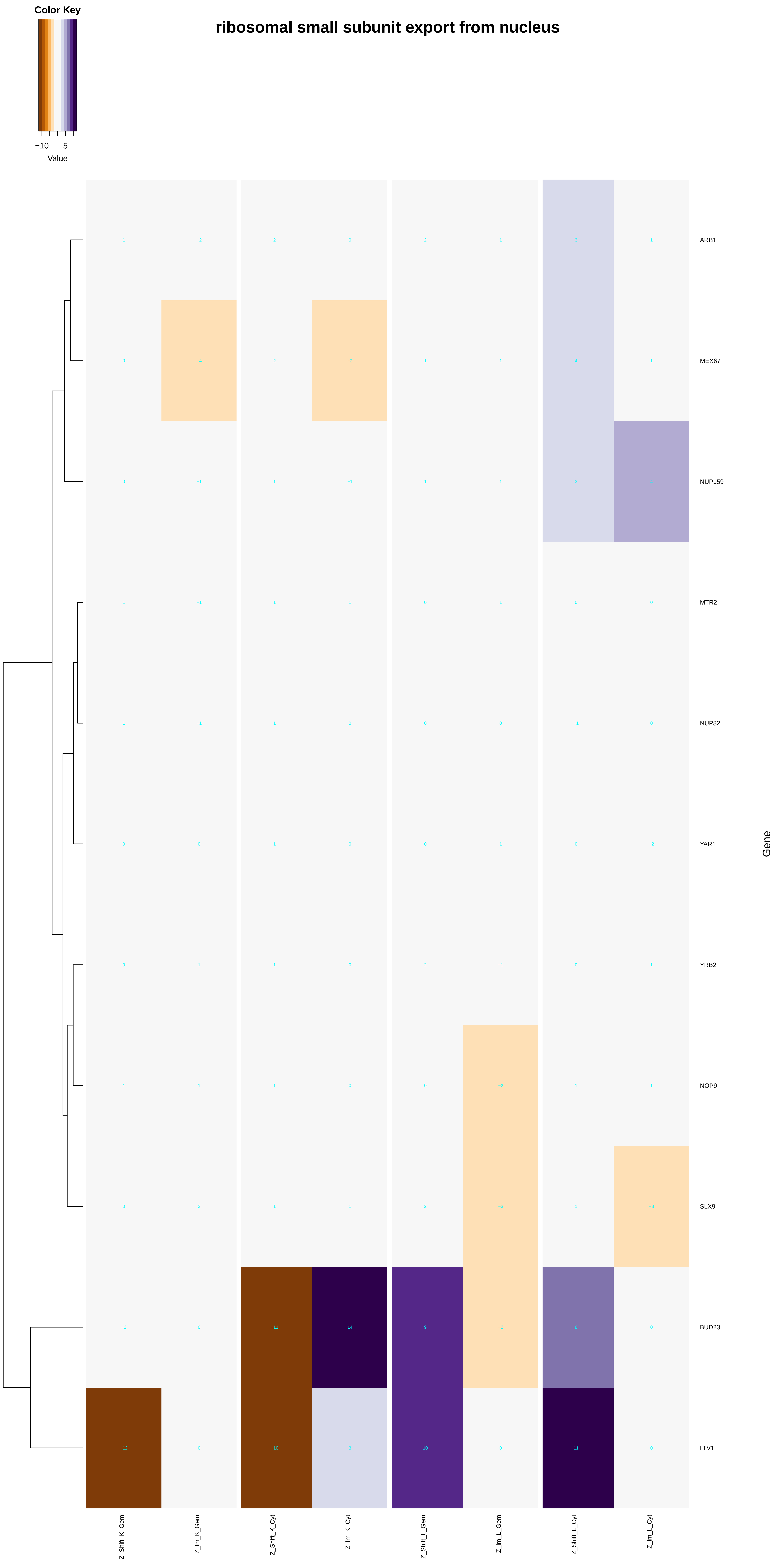

Color Key
−10
5
Value
ribosomal small subunit export from nucleus
ARB1
MEX67
NUP159
MTR2
NUP82
YAR1
YRB2
NOP9
SLX9
BUD23
LTV1
Gene
Z_Shift_K_Gem
Z_Im_K_Gem
Z_Shift_K_Cyt
Z_Im_K_Cyt
Z_Shift_L_Gem
Z_Im_L_Gem
Z_Shift_L_Cyt
Z_Im_L_Cyt
